# Supplementary material for: Experiences of oncology researchers in the Veterans Health Administration during the COVID-19 pandemic
Source: PLoS One. 2024 Jan 24;19(1):e0290785. doi: 10.1371/journal.pone.0290785 (PMC10807772; doi:10.1371/journal.pone.0290785)
Supplement: S1 File — (PDF) [file pone.0290785.s001.pdf]

# Research Experience During the COVID-19 Pandemic at Centers of Excellence within the Veterans Health Administration

Please complete the survey below to the best of your ability.

Thank you!

## Research Role and Research Focus

Select your role(s) prior to COVID-19 pandemic:

- ☐ Medical Oncologist
- ☐ Urologist
- ☐ Clinical Investigator
- ☐ Data Analyst
- ☐ Post-Doctoral Researcher
- ☐ Administrator
- ☐ Clinical Trainee
- ☐ Research Trainee
- ☐ Research Coordinator
- ☐ Research Assistant
- ☐ Research Nurse
- ☐ Research Pharmacist

I have a diverse range of research interests.

- ◯ Strongly Disagree
- ◯ Disagree
- ◯ Neutral
- ◯ Agree
- ◯ Strongly Agree

I feel that I was able to focus on my active research during the COVID-19 pandemic.

- ◯ Strongly Disagree
- ◯ Disagree
- ◯ Neutral
- ◯ Agree
- ◯ Strongly Agree

Describe your area of research prior to COVID-19 pandemic.

__________________________________

## Research During COVID-19

My research focus changed during the COVID-19 pandemic.

- ◯ Strongly Disagree
- ◯ Disagree
- ◯ Neutral
- ◯ Agree
- ◯ Strongly Agree

Describe how your research focus changed during the COVID-19 pandemic.

__________________________________________

Describe your research goals during the COVID-19 pandemic.

__________________________________________

| | |
|---|---|
| I achieved my research goals during the COVID-19 pandemic. | ◯ Strongly Disagree<br>◯ Disagree<br>◯ Neutral<br>◯ Agree<br>◯ Strongly Agree |
| I gained new research skills during the COVID-19 pandemic. | ◯ Strongly Disagree<br>◯ Disagree<br>◯ Neutral<br>◯ Agree<br>◯ Strongly Agree |
| I found research difficult during the COVID-19 pandemic challenging. | ◯ Strongly Disagree<br>◯ Disagree<br>◯ Neutral<br>◯ Agree<br>◯ Strongly Agree |
| I felt personal responsibility to research COVID-19. | ◯ Strongly Disagree<br>◯ Disagree<br>◯ Neutral<br>◯ Agree<br>◯ Strongly Agree |
| I felt influenced by my peers to change my research focus to a COVID-19 related topic during the pandemic. | ◯ Strongly Disagree<br>◯ Disagree<br>◯ Neutral<br>◯ Agree<br>◯ Strongly Agree |
| I felt influenced by the public to change my research focus during the COVID-19 pandemic. | ◯ Strongly Disagree<br>◯ Disagree<br>◯ Neutral<br>◯ Agree<br>◯ Strongly Agree |
| I felt influenced by the VA to change my research focus during the COVID-19 pandemic | ◯ Strongly Disagree<br>◯ Disagree<br>◯ Neutral<br>◯ Agree<br>◯ Strongly Agree |
| I felt supported to change my research focus during the COVID-19 pandemic. | ◯ Strongly Disagree<br>◯ Disagree<br>◯ Neutral<br>◯ Agree<br>◯ Strongly Agree |
| Describe support structures that were helpful. | __________________________________________ |
| Describe ways in which support felt lacking. | __________________________________________ |

| | |
|---|---|
| My other research projects were compromised by COVID-19. | ◯ Strongly Disagree<br>◯ Disagree<br>◯ Neutral<br>◯ Agree<br>◯ Strongly Agree |
| Prior to the COVID-19 pandemic my institution had active investigators focused on infectious disease research. | ◯ Strongly Disagree<br>◯ Disagree<br>◯ Neutral<br>◯ Agree<br>◯ Strongly Agree |
| How do you feel your research resources or support were affected by the COVID-19 pandemic? | __________________________________ |
| My institution has appropriate resources to support COVID-19 research. | ◯ Strongly Disagree<br>◯ Disagree<br>◯ Neutral<br>◯ Agree<br>◯ Strongly Agree |
| Clinical responsibilities during the COVID-19 pandemic interfered with my research project. | ◯ Strongly Disagree<br>◯ Disagree<br>◯ Neutral<br>◯ Agree<br>◯ Strongly Agree |

## Personal Attributes

| | |
|---|---|
| Personal circumstances were influential in my work during the COVID-19 pandemic. | ◯ Strongly Disagree<br>◯ Disagree<br>◯ Neutral<br>◯ Agree<br>◯ Strongly Agree |
| Describe how personal circumstances limited or facilitated your research during  the COVID-19 pandemic. | __________________________________ |
| Describe how the COVID-19 pandemic changed your belief about the role of research. | __________________________________ |
| Emotions motivated me in my COVID-19 research. | ◯ Strongly Disagree<br>◯ Disagree<br>◯ Neutral<br>◯ Agree<br>◯ Strongly Agree |
| Emotions limited me in my COVID-19 research. | ◯ Strongly Disagree<br>◯ Disagree<br>◯ Neutral<br>◯ Agree<br>◯ Strongly Agree |

| | |
|---|---|
| I feel that I am often able to adapt to difficult situations. | ◯ Strongly Disagree<br>◯ Disagree<br>◯ Neutral<br>◯ Agree<br>◯ Strongly Agree |
| I felt personal health risk about performing COVID-19 related research. | ◯ Strongly Disagree<br>◯ Disagree<br>◯ Neutral<br>◯ Agree<br>◯ Strongly Agree |
| I believe I am able to make a meaningful contribution to improving the COVID-19 pandemic. | ◯ Strongly Disagree<br>◯ Disagree<br>◯ Neutral<br>◯ Agree<br>◯ Strongly Agree |
| I believe I have gained a better understanding about public health during the COVID-19 pandemic. | ◯ Strongly Disagree<br>◯ Disagree<br>◯ Neutral<br>◯ Agree<br>◯ Strongly Agree |
| I felt like I had a good baseline knowledge of research methods applicable to COVID-19 research. | ◯ Strongly Disagree<br>◯ Disagree<br>◯ Neutral<br>◯ Agree<br>◯ Strongly Agree |
| I felt personally rewarded for pursuing research related to COVID-19. | ◯ Strongly Disagree<br>◯ Disagree<br>◯ Neutral<br>◯ Agree<br>◯ Strongly Agree |
| I felt personally penalized for pursuing research related to COVID-19. | ◯ Strongly Disagree<br>◯ Disagree<br>◯ Neutral<br>◯ Agree<br>◯ Strongly Agree |
| I felt professionally rewarded for pursuing research related to COVID-19. | ◯ Strongly Disagree<br>◯ Disagree<br>◯ Neutral<br>◯ Agree<br>◯ Strongly Agree |
| I felt professionally penalized for pursuing research related to COVID-19. | ◯ Strongly Disagree<br>◯ Disagree<br>◯ Neutral<br>◯ Agree<br>◯ Strongly Agree |
| I had experience with infectious disease research prior to the COVID-19 pandemic. | ◯ Strongly Disagree<br>◯ Disagree<br>◯ Neutral<br>◯ Agree<br>◯ Strongly Agree |

## Future Research Plans

I have a plan to return or have already returned to my prior research focus.

- ◯ Strongly Disagree
- ◯ Disagree
- ◯ Neutral
- ◯ Agree
- ◯ Strongly Agree

I intend to incorporate features of my COVID-19 related research into my research career.

- ◯ Strongly Disagree
- ◯ Disagree
- ◯ Neutral
- ◯ Agree
- ◯ Strongly Agree

I believe redistribution of research resources to COVID-19 related research will have an impact on cancer research

- ◯ Strongly Disagree
- ◯ Disagree
- ◯ Neutral
- ◯ Agree
- ◯ Strongly Agree

I feel I will be able to respond to future crises in my research career.

- ◯ Strongly Disagree
- ◯ Disagree
- ◯ Neutral
- ◯ Agree
- ◯ Strongly Agree

I think my research during the COVID-19 pandemic makes me more competitive for future research positions.

- ◯ Strongly Disagree
- ◯ Disagree
- ◯ Neutral
- ◯ Agree
- ◯ Strongly Agree

How do you think the COVID-19 pandemic will change the utilization and distribution of research resources?

______________________________________

## Demographics

| | |
|---|---|
| What state do you work in? | ◯ Alabama ◯ Alaska<br>◯ Arizona ◯ Arkansas<br>◯ California ◯ Colorado<br>◯ Connecticut ◯ Delaware<br>◯ Florida ◯ Georgia<br>◯ Hawaii ◯ Idaho ◯ Illinois<br>◯ Indiana ◯ Iowa ◯ Kansas<br>◯ Kentucky ◯ Louisiana<br>◯ Maine ◯ Maryland<br>◯ Massachusetts ◯ Michigan<br>◯ Minnesota ◯ Mississippi<br>◯ Missouri ◯ Montana<br>◯ Nebraska ◯ Nevada<br>◯ New Hampshire ◯ New Jersey<br>◯ New Mexico ◯ New York<br>◯ North Carolina ◯ North Dakota<br>◯ Ohio ◯ Oklahoma<br>◯ Oregon ◯ Pennsylvania<br>◯ Rhode Island ◯ South Carolina<br>◯ South Dakota ◯ Tennessee<br>◯ Texas ◯ Utah ◯ Vermont<br>◯ Virginia ◯ Washington<br>◯ West Virginia ◯ Wisconsin<br>◯ Wyoming ◯ District of Columbia (Washington, DC) |
| What is your age range? | ◯ 20-30<br>◯ 30-40<br>◯ 40-50<br>◯ 50-60<br>◯ 60-70<br>◯ 70+ |
| How do you identify? | ◯ Male<br>◯ Female<br>◯ Nonbinary<br>◯ Prefer not to answer |
| What professional degrees do you hold? | ______________________________ |
| Do you hold a leadership role at your institution? | ◯ Yes ◯ No |
| How many years have you been working in your current position? | ______________________________ |
