# Supplementary material for: Experiences of oncology researchers in the Veterans Health Administration during the COVID-19 pandemic
Source: PLoS One. 2024 Jan 24;19(1):e0290785. doi: 10.1371/journal.pone.0290785 (PMC10807772; doi:10.1371/journal.pone.0290785)
Supplement: Attachment — Submitted filename: Response to Reviewers.docx [file pone.0290785.s002.docx]

May 19, 2023

Dear Editors,

We thank the reviewers for their comments on our manuscript and have edited the manuscript to address their concerns. Points raised by the Academic Editor and reviewers (italicized) are addressed below:

Academic Editor:

*Please ensure that you have submitted a justification for the chosen sample size.*

We have included additional information regarding how the sample population was identified in the Methods section. These researchers were identified by via two separate research contacts lists, representing participants in national VA lung and prostate cancer consortia, to ensure that respondents were actively involved in oncology related research.

*Please ensure that you included the measures taken that the low response rate did not cause any bias*

The low response rate of this survey is discussed as the main limitation of this study in the updated Discussion section.

Journal Requirements:

A full ethics statement is now included in the Methods section.

Copyrighted images are removed from Figure 2.

Reviewer 1:

*- The manuscript tackles an important issue and the authors used sound methods to obtain data.*

*Abstract:*

*- Concise and informative*

*Introduction:*

*- Provides a convenient answer to what is known and what is not known and the implication of the study*

*Methods:*

*- Well written with relevant details*

*Results:*

*- Too long section (8 pages).*

*- This could be reduced to 2 pages or even less if the authors highlighted only the main findings and refer the reader to the table or to the figures.*

The results section has been shortened, to highlight only selected main quantitative findings, with the remainder of the results presented in Table 3.

*- In table 1, there is an overlap between age groups e.g. from 20-30 and then from 30-40; where to put a patient with an age of 30 years?*

The age ranges in Table 1 have been corrected to avoid overlap.

*Discussion:*

*- Discussion is expected to be one unit with paragraphs; each to cover an objective related information. Rewrite*

*- Avoid putting with subtitles. Remove subtitle in lines 384, 367, 385*

Subtitles in the Discussion sections have been removed. The Discussion has been rewritten to highlight where this study fits into prior related studies.

*- There are 2 different reference styles in the discussion (line 310, 312 vs 315, 318)*

Reference styles have been corrected.

*- The authors missed to state the limitations. Low response rate is the main limitation.*

The low response rate has been highlighted as a limitation of this study.

*Others:*

*- No title seen for figure 1 and 2.*

*- Figures are of poor quality; redesign*

Figures have been redesigned and titles included with separately submitted figures.

Reviewer 2:

*1. Research title can be more specific to oncology researchers*

Title has been updated to reflect this study was specific to oncology researchers.

*2. Abstract-conclusion: too general. Please write more specifically for this paper.*

Specific findings and conclusions from this study are included in the abstract conclusion.

*3. Abstract-would be better to add keywords*

Unsure of how to incorporate keywords into the abstract.

*4. Introduction: What is novelty in research, statement of problem is not clear.*

Statement of problem and novelty included in Introduction lines 86-89, 95-98.

*5. Materials and methods -Unclear calculation of the sample size, how many clinical researchers, faculty were there in that area, and based on that number, calculate the total size (only 118???). Only 43 respondents? These can be preliminary results or a pilot study for this.*

We have included additional information regarding how the sample population was identified in the Methods section.

*6. Response rate was only 36%, what could be the possible reasons and what initiatives were taken to improve participation.*

Low response rate discussed as limitation of this study in Discussion section.

*7. Table heading must be on top not at the bottom. Table 1 age categories are confusing, where age group 30 comes under two categories…same goes to other age categories, please check and fix.*

Table headings changed to top and age categories have been corrected.

*8. Figure 1 is not clear so better to modify in excel*

Figure 1 updated and resized to be clearer.

*9. Results are way too long and must be summarized and presented in a concise manner.*

Results section has been shortened.

*10. The discussion needs to be more specific, say more about the obtained results and explain them in the context of similar studies.*

The Discussion section has been rewritten to include information about similar studies for context.

*11. What are the strengths and limitations of this study?*

Limitations are now included in Discussion section.

*12. The conclusion needs to be more specific.*

Discussion and conclusion rewritten to highlight more specific findings and relevance of this study.

*13. The literature needs to be adequately cited in the discussion.*

Citations added to Discussion.

We hope that the revisions in our manuscript will be sufficient to make our manuscript suitable for publication.

Sincerely,

Kenneth Csehak
